# Grading operative findings at laparoscopic cholecystectomy following the new scoring system in Duhok governorate: Cross sectional study

**DOI:** 10.1016/j.amsu.2020.10.035

**Published:** 2020-10-23

**Authors:** Sanar Majeed Jameel, Muwafaq Masoud Bahaddin, Ayad Ahmad Mohammed

**Affiliations:** a Senior House Officer in General Surgery, Azadi Teaching Hospital, Directorate General of Health, DUHOK, Kurdistan Region, Iraq; b Department of Surgery, College of Medicine, University of Duhok, DUHOK, Kurdistan Region, Iraq

**Keywords:** Laparoscopic cholecystectomy, Open cholecystectomy, Gallstones, Difficult cholecystectomy, Intraoperative scoring system, Gallbladder adhesions

## Abstract

**Introduction:**

Numerous preoperative scoring systems predict difficult laparoscopic cholecystectomy. Recently, the intraoperative difficulties which are facing surgeons are studied. A new scoring system categorize patients according to many intraoperative findings with a final outcome whether converting to open cholecystectomy or continuing laparoscopically.

**Patients and methods:**

This prospective study included 120 patients admitted for laparoscopic cholecystectomy for symptomatic gallstones from October 2019 to August 2020. Intraoperative difficulties were evaluated and patients were categorized according to intraoperative scoring for cholecystitis severity and compared depending to the rate of conversion to the open technique.

**Results:**

Most patient were middle aged females having multiple gallstones, the mean operation time was 35 min and 7.8% of patients were converted to open cholecystectomy because of intraoperative difficulty.

There was a significant correlation between the conversion rate and each of distended and/or contracted gall bladder, inability to grasp the gall bladder with traumatic forceps, stone ≥1 cm impacted in Hartman's pouch, and bile or pus outside gallbladder (P values: 0.002, 0.000, 0.008 and 0.015) respectively, and no significant correlation with gallbladder adhesions, adhesions from previous upper abdominal surgery, BMI>30, and Time to identify cystic artery and duct >90 min (P values: 0.123, 1, 1, 0.078) respectively.

**Conclusion:**

New intraoperative scoring systems are valuable in predicting difficulties and preventing increase operation time and possible injuries. The main points of difficulties are distended or contracted gallbladder, large stone impaction, difficult grasping the wall of the gall bladder and the presence of bile or pus outside the gall bladder.

## Highlights

• Laparoscopic cholecystectomy is a safe procedure with some difficulties.
• The definition of difficult LC is not well established until now.
• Recently intraoperative difficulties facing the surgeons are studied.
• Inflammation, adhesions, and obesity are some factors for difficulty.

## 1 Introduction

Gall stones and their complications are one of the most common indications for both elective and emergency surgeries whether laparoscopic or open surgery. Laparoscopic cholecystectomy (LC) was first done in 1985, since that time it has widely replaced open cholecystectomy as the standard of care for symptomatic gall stones [[1], [2], [3], [4], [5]].

The procedure of LC is usually performed by the conventional 4 ports technique, but some modifications of the technique may be done depending on the surgeon's experience and the hospital protocols [4,6].

LC is a relatively safe procedure and very effective. Surgeons may encounter some difficulties during the procedure starting from intraperitoneal access, achieving pneumoperitoneum, releasing adhesions, and identification of the proper anatomy. Identification of the anatomy during surgery which is arranged in a mirror image pattern particularly the critical view of safety is the most crucial step during LC. Many complications are reported during the procedure like biliary and vascular injuries, port site complications and injuries due to adhesions [3,4,7].

The definition of difficult LC is not well established until now, and it may be very difficult to be established, this is because the difficulty is depend not only on patient's factors but also on the surgeon's experience and skills. Several factors are widely accepted for the difficulties such as the presence of inflammation, adhesions, and obesity. The adhesions may be due to previous upper abdominal operations or adhesions with the gall bladder due to attacks of inflammation [8].

There are numerous preoperative scoring systems which are aiming to predict difficult surgeries depending on various anatomical, imaging and laboratory findings, in the contrary there are few intraoperative scoring systems which predict the intraoperative difficulties. Recently some authors studied the main points of difficulties facing the surgeons during LC. A new scoring system was placed recently at 2015 aiming to categorize patients according to many intraoperative findings with a final outcome whether or not to convert the operation to open cholecystectomy or continuing with the laparoscopic approach [1].

## 2 Patients and methods

This is a prospective study which included 120 patients who were admitted for laparoscopic cholecystectomy for symptomatic gall stones, patients were randomly recruited in this study. The operations were done by 2 general surgeons who are experienced in the field of laparoscopic surgery. Patients were enrolled from 2 surgical centers within the period from October 2019 to August 2020. Patients with obstructive jaundice, malignancy, and those who refused to be enrolled in this study were excluded, one patient with a midline gallbladder was also excluded. An informed consent was obtained from each patient to be enrolled in the current study. Intraoperative findings were then collected and patients were categorized using the intraoperative scoring for cholecystitis severity which is recommended according to a large meta-analysis study from published articles between 1965 and 2014, Table 1 [2].

**Table 1:** Operative scoring system for cholecystitis severity.

Operative grading system	Score
***Gall bladder appearance*** No Adhesions Adhesions <50% of GB Adhesions burying GB ***Maximum***	0 1 3 3
***Distension/Contraction*** Distended/Contracted gall bladder Unable to grasp with a traumatic forceps Stone ≥1 cm impacted in Hartman's Pouch	1 1 1
***Access*** BMI >30 Adhesions previous surgery limiting access	1 1
***Severe sepsis/complications*** Bile or Pus outside GB Time to identify cystic artery and duct >90 min	1 1
**Total score**	**10**

Patients then were categorized on the bases whether they were converted to open cholecystectomy or not into 2 groups and the comparison were performed regarding the intraoperative scoring system of difficulties.

## 3 Statistical analyses

Categorical variables were described in frequencies and percentage, while continuous variables were described in means and standard deviations. P values of less than 0.05 were considered significant. The data were analyzed using the Statistical Package for Social Sciences (SPSS 25, IBM: USA).

## 4 Research registration

The study gained approval of the ethical committee of the Kurdistan Board for Medical Specializations according to the registration number 628 at September 2020.

The research is registered according the World Medical Association's Declaration of Helsinki 2013 at the research registry at the 30th of August 2020, Research registry UIN: research registry **5964**.

The work of this article has been reported in line with the STROCSS criteria [9].

## 5 Results

**Fig. 1:**
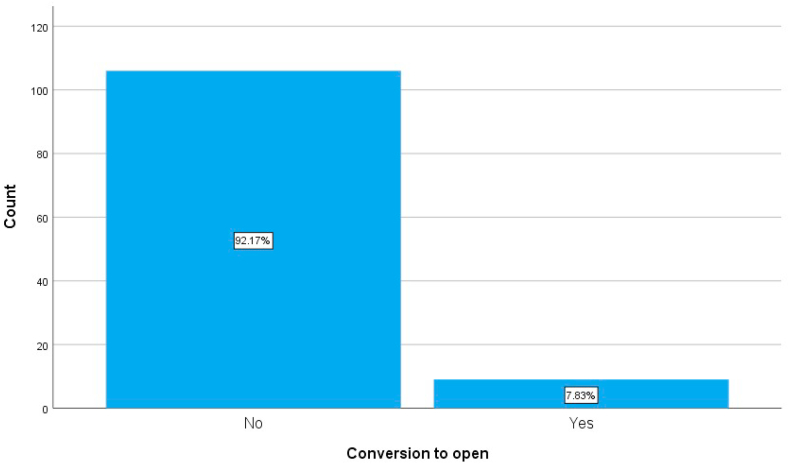
A simple bar chart showing the percentage of both groups of patients whether they were converted or not to open cholecystectomy.

Most patient in our study were middle aged females and had no history of hospitalization due to gall stones. The majority had multiple gall stones, the mean operation time was 35 min and 7.8% of patients were converted to open cholecystectomy because of intraoperative difficulties. Table 2 and Fig. 1.

**Table 2:** The general characteristics of the patients involved in this study.

Main category	Subcategories	Frequency	Percentage
Age of the patient **(M;SD)** Range: **17**–**75**		42.68	12.519
Gender	Female Male	85 30	73.9 26.1
History of previous hospitalization	No admissions Biliary colic Acute cholecystitis	62 24 29	53.9 20.9 25.2
Comorbidities*	No comorbidities Comorbidities	99 16	86.1 13.9
History of jaundice	No history of jaundice History of jaundice	107 8	93.0 7.0
Number of stones	Single stone Multiple stones	20 95	17.4 82.6
Total operation time in minutes **(M;SD)** Range: **15**–**90**		35.03	15.972
Conversion to open	No Yes	106 9	92.2 7.8

*Like diabetes mellitus, hypertension, ischemic heart diseases.

According to the intraoperative score categorization, 45.2% of our patients were categorized as mild core, and only 0.9% had the extreme score for difficulty. Table 3.

**Table 3:** Intra Operative Scores/Categories of the involved patients.

Intra Operative Scores/Categories	Frequency	Percent
Less than 2 (Mild)	52	45.2
2–4 (Moderate)	44	38.3
5–7 (Very difficult)	18	15.7
8–10 (Extreme)	1	0.9

**Fig. 2:**
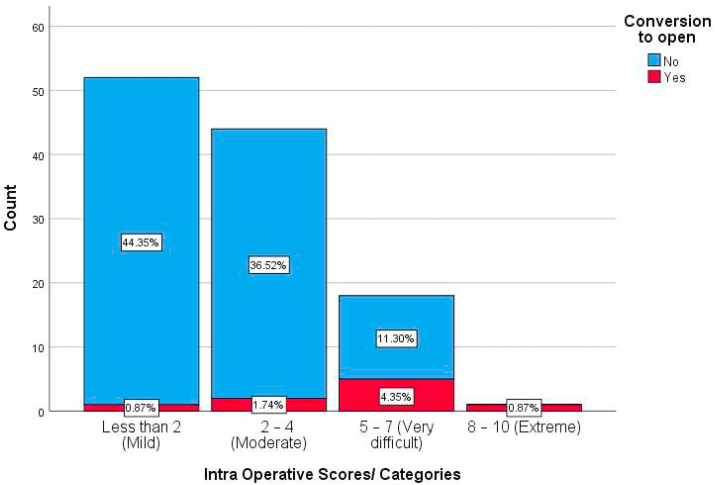
A stacked bar chart showing the Intra Operative Scores/Categories in both groups of patient whether converted or not converted to open cholecystectomy.

The percentages of each category of both groups are illustrated in Fig. 2.

The most significant correlations were found with distended and/or contracted gall bladder, inability to grasp the wall with a traumatic forceps, an impacted stone in the Hartman's pouch, and pus or bile outside the gall bladder. No correlation was detected with other findings. The correlations were calculated using the Fisher's exact test. Table 4.

**Table 4:** Comparison between patients converted to open and those not converted to open cholecystectomy adopting Intra-operative New Scoring System for cholecystitis severity.

Intra Operative Findings	Conversion to open		Sig. (2-sided)
	No (n = 9)	Yes (n = 112)	
Gallbladder appearance **No Adhesions** **Adhesions** < **50% of GB** **Adhesions burying GB**	54 (50.9%) 31 (29.2%) 21 (19.8%)	2 (22.2%) 3 (33.3%) 4 (44.4%0	0.123
Distended and/or contracted shriveled gall bladder **No** **Yes**	69 (65.1%) 37 (34.9%)	1 (11.1%) 8 (88.9%)	**0.002**
Unable to grasp with a traumatic forceps **No** **Yes**	93 (87.7%) 13 (12.3%)	1 (11.1%) 8 (88.9%)	**0.000**
Stone ≥1 cm impacted in Hartman's Pouch **No** **Yes**	91 (85.8%) 15 (14.2%)	4 (44.4%) 5 (55.6%)	**0.008**
BMI > 30 **No** **Yes**	67 (63.2%) 39 (36.8%)	6 (66.7%) 3 (33.3%)	1.00
Adhesions previous surgery limiting access **No** **Yes**	98 (92.5%) 8 (7.5%)	9 (100.0%) 0 (0.0%)	1.00
Bile or Pus outside GB **No** **Yes**	101 (95.3%) 5 (4.7%)	6 (66.7%) 3 (33.3%)	**0.015**
Time to identify cystic artery and duct >90 min **No** **Yes**	106 (100.0%) 0 (0.0%)	8 (88.9%) 1 (11.1%)	0.078

Each category of severity was compared in both groups, the correlation was very significant between the two groups of patients. Table 5.

**Table 5:** Comparison of Intraoperative Scores/Categories between patients converted to open and those not converted to open cholecystectomy adopting Intra-operative New Scoring System for cholecystitis severity.

Intraoperative Scores/Categories	Conversion to open		Total (n = 120)	Sig. (2-sided)
	No (n = 9)	Yes (n = 112)		
Less than 2 (Mild) 2–4 (Moderate) 5–7 (Very difficult) 8–10 (Extreme)	51 (48.1%) 42 (39.6%) 13 (12.3%) 0 (0.0%)	1 (11.1%) 2 (22.2%) 5 (55.6%) 1 (11.1%)	52 (45.2%) 44 (38.3%) 18 (15.7%) 1 (0.9%)	0.000

## 6 Discussion

Difficult LC is a real challenge which face surgeons during surgery and many times it is unpredictable before surgery and discovered only intraoperatively. Understanding the anatomy of the biliary system and the laparoscopic principles are among the best ways to optimize performance during laparoscopic cholecystectomy, proper positioning of the patient and correct port placement are other factors. Although LC is one of the most widely practiced surgical procedures, it is still associated with some morbidity and even mortality [2,7,8].

Many points of difficulties were studied by the authors, one of the most important points is the BMI, it is agreed that BMI > than 30 is regarded as one of the points of difficulties, in our study there was no significant correlation with the BMI and the conversion rate (P value 1). To the contrary of our finding, authors considered BMI greater than 30 as one of the points which was associated with higher conversion rates [3,10,11].

Most of our patients were young and middle aged females, and more than have of them had no previous history of hospitalization due to gall stones. About 82% of patients had multiple stones. In some literature male gender is mentioned to be one of the points of difficulty, and the presence of large stones is also among the points of difficulties. Sometimes a large stone may be impacted in the Hartman's pouch making identification of the cystic duct and artery difficult and increase the operation time, in our study the presence of a single stone ≥1 cm impacted in Hartman's pouch was one of the main points of difficulty and the correlation with the rate of conversion was significant (P value 0.008), this point may be managed by gentle pushing of the stone upward toward the fundus of the gall bladder and application of the grasping forceps below the stone, then pushing the gall bladder toward the right shoulder [10,12].

Operative time, especially the time to identify the cystic duct and artery intraoperatively, is one of the critical steps during surgery, this time depends greatly on the surgeon's experience, the presence of acute inflammation, stone impaction at the Hartman's pouch, and the presence of some anomalies of the biliary tree. In our study the mean operative time was around 35 min and the time to identify the cystic duct and artery had no significant correlation with the conversion rate (P value 0.078). Many authors documented similar operative time in their published articles, sometimes when the anatomy is very clear and the artery and the cystic duct are obvious the times will be shorter [3,4,13,14].

The conversion rate to the open surgery is ranging from 1 to 13% in most articles, in our patients the conversion rate was 7.83%, this is regarded as acceptable rate compared to the literature [15].

The presence of acute cholecystitis is associated with increase wall thickness, similarly chronic cholecystitis may be associated with increase was thickness and shriveled gall bladder and both are regarded as points of intraoperative difficulties, in our study there was a very significant correlation between the conversion rate and both distended and/or contracted gall bladder and the inability to grasp with a traumatic forceps (P values 0.002 and 0.000) respectively, increase wall thickness is associated with higher rates of conversion to the open technique because of technical difficulties [8,16,17].

The presence of adhesions will limit the view and make dissection difficult especially dense adhesions. Adhesions due to upper abdominal operations are the main concern during laparoscopic cholecystectomy. Another concern for the surgeons is that adhesions due to previous surgery for hernia in the umbilical region, this make difficulty during intra-abdominal access, entering the abdomen adopting the open technique instead of the closed technique using the verses needle or changing the port site to regions away from the previous scar will greatly reduce the risk of injuries and will make safer intra-peritoneal access. In our study the presence of adhesions due to upper abdominal surgery was not significantly associated with the conversion rate (P value 1), some studies concluded that it may be associated with increase operation time with no higher rates of conversion [12,16,18,19].

Other types of adhesions are those between the gall bladder and the omentum or bowel, this is due to attacks of previous cholecystitis which form inflammatory reaction, sometimes the adhesions may be limited to some parts of the gall bladder or the whole gall bladder may be obscured by adhesions. In our study the association with gall bladder adhesions was not significant (P value 0.123), most patients in our study had no adhesions with the gall bladder, while adhesions which buried the gallbladder completely were present in around 25% of our patients, adhesions in the region of the cystohepatic (Calot's triangle) triangle have been found to be the most difficult ones [10,[20], [21], [22]].

Intraoperative detection of free pus or bile outside the gall bladder may indicate either gangrenous or perforated gall bladder and may be associated with higher conversion rates, in our study the association was also significant (P value 0.015), sometimes this collection may be detected even preoperatively using different imaging techniques [10].

## 7 Conclusion

New intraoperative scoring systems are valuable in predicting difficulties and preventing increase the operation time and possible injuries, the main points of difficulties are distended or contracted gallbladder, large stone impaction, difficult grasping the wall of the gall bladder and the presence of bile or pus outside the gall bladder.

## Funding

No source of funding other than the authors.

## Author contribution

Dr Sanar Majeed Jameel did the data collection.

The concept of the study was done by Dr Muwafaq Masoud Bahaddin.

Study design, analysis, and writing is done by Dr Ayad Ahmad Mohammed.

Final approval of the manuscript is done Dr Ayad Ahmad Mohammed.

## Research registration unique identifying number (UIN)

Researchregistry **5964**

https://www.researchregistry.com/browse-the-registry#home/?view_2_search = 5964&view_2_page = 1.

## Guarantor

Dr Ayad Ahmad Mohammed.

## Provenance and peer review

Not commissioned, externally peer reviewed.

## Declaration of competing interest

There is no conflict of interest to be declared.
